# Blinded by the load: attention, awareness and the role of perceptual load

**DOI:** 10.1098/rstb.2013.0205

**Published:** 2014-05-05

**Authors:** Nilli Lavie, Diane M. Beck, Nikos Konstantinou

**Affiliations:** 1 Institute of Cognitive Neuroscience, University College London, London, UK; 2 Department of Psychology and Beckman Institute, University of Illinois, Urbana-Champaign, IL, USA; 3 Center for Applied Neuroscience, University of Cyprus, Nicosia, Cyprus

**Keywords:** attention, conscious visual awareness, perceptual load, change blindness, inattentional blindness, contrast response function

## Abstract

What is the relationship between attention and conscious awareness? Awareness sometimes appears to be restricted to the contents of focused attention, yet at other times irrelevant distractors will dominate awareness. This contradictory relationship has also been reflected in an abundance of discrepant research findings leading to an enduring controversy in cognitive psychology. Lavie's load theory of attention suggests that the puzzle can be solved by considering the role of perceptual load. Although distractors will intrude upon awareness in conditions of low load, awareness will be restricted to the content of focused attention when the attended information involves high perceptual load. Here, we review recent evidence for this proposal with an emphasis on the various subjective blindness phenomena, and their neural correlates, induced by conditions of high perceptual load. We also present novel findings that clarify the role of attention in the response to stimulus contrast. Overall, this article demonstrates a critical role for perceptual load across the spectrum of perceptual processes leading to awareness, from the very early sensory responses related to contrast detection to explicit recognition of semantic content.

## 1. Introduction

### (a) Definitions and historical context

The terms attention and awareness have acquired various meanings over years of intensive study of both. It is thus useful to clarify our use of these terms. Our use of the term attention refers to the allocation of limited-capacity mental resources to processing. Our use of the term awareness refers to visual or perceptual awareness: the phenomenal experience related to perception that is accessible for report.

Our focus, however, is not on one definition or another but rather on the relationship between attention and awareness. At times, attention and awareness appear intimately linked in our personal experience, as when we find ourselves solely aware of what we are attending to (e.g. a football match) while being oblivious to what we do not attend to (e.g. our friend talking to us). At other times, attention and awareness appear to diverge, and we find ourselves unable to block awareness of irrelevant distractions despite focusing attention on our task (e.g. being distracted by the indication of a new email while reading this article).

Psychology research has mirrored this puzzling pattern. Indeed, the very question of whether awareness depends on paying attention at all has stimulated a heated controversy that lasted several decades. Demonstrations that people failed to note various types of unattended events, including those that would appear to be rather conspicuous (e.g. a woman crossing a game pitch, holding an open umbrella, while people attend to the ball game on the pitch [1]), have led to the early selection view, in which selective attention filters information early on in the processing stream, on the basis of rudimentary physical characteristics of the stimuli, before full perception and awareness of the stimulus meaning can occur. In this view, awareness clearly depends on the allocation of focused attention. The early selection view could not account, however, for other findings that demonstrated intrusions of unattended information into awareness [2,3]. Such findings led to the opposing late selection view in which attentional selection occurs later in the processing stream, after full perceptual awareness, filtering out irrelevant information from processes such as memory and overt responses [4]. Under this view, failures to note unattended events, which provided support for the early selection view, merely reflected failures of memory rather than perception. Such an account was particularly pertinent as, in most paradigms, participants' reports were collected retrospectively, after the unattended event. Lending support to the early selection view, however, Treisman & Geffen [5] had participants respond immediately to both attended and unattended events (by tapping on the table when targets are presented) but nonetheless found that while participants detected 87% of the attended target words, they only detected 8% of unattended targets. While experiments such as these would provide support for one or the other theory, no consistent pattern emerged. Thus, while the early versus late selection debate stimulated much research, it remained unresolved for many decades (for reviews, see [6–8]).

### (b) Load theory

Load theory of attention [9,10] has offered a resolution to this debate by viewing the question in a different light, applying a capacity approach which has been rather overlooked in previous theories of selective attention (for discussion, see [8]) to understand the relationship between selective attention and perception. According to load theory, perceptual processing has limited capacity but proceeds automatically in an involuntary, mandatory manner on all information within its capacity. It follows, therefore, that in attended tasks involving a large amount of information, in other words high perceptual load, capacity is fully exhausted by the processing of the attended information, resulting in no perception of unattended information. By contrast, in tasks of low perceptual load, because perception cannot be voluntarily stopped, spare capacity from processing the information in the attended task will inevitably spill over, resulting in the perception of task-irrelevant information that people intended to ignore.

Effective selective and focused attention therefore requires not just active maintenance of a top-down attentional priority for a task-relevant set (e.g. as indicated in the task instructions) but also a high level of perceptual load that will tax all the available capacity (e.g. [11]). While clear top-down settings are necessary to distinguish relevant from irrelevant information so that higher priority is given to the relevant information, prevention of capacity allocation (spillover) to the irrelevant information can only occur as a natural consequence of reduced availability of perceptual processing capacity under load.^1^

Load theory not only provides a resolution to the early versus late selection debate, but also clarifies the nature of attention in a theory that allows attention mechanisms to be more fully integrated with theories of awareness. The claim that all perceptual processing has limited capacity refers to both conscious and unconscious processing. Thus, the effects of load are not confined to a specific level of processing: for example, not just to accessibility for report. However, load theory does have clear implications for awareness. For a stimulus to reach awareness, it needs to receive sufficient processing capacity for its content (e.g. a vertical line over a dot) and meaning (an exclamation mark) to be perceived. Awareness will thus be clearly confined to just the attended task information under conditions of high perceptual load (allowing for early selection effects of top-down attentional selection). In conditions of low load, however, awareness will not be confined to just those stimuli specified by top-down selection settings as ‘to be attended to’. Owing to the involuntary nature of perception, irrelevant information that people intend to ignore can reach full awareness under conditions of low perceptual load (resulting in late attentional selection). Load theory thus makes clear predictions for the effects of attention on awareness: awareness will depend on the level of perceptual load of the attended processing.

Note that with respect to the perennial issue of the relationship between attention and awareness, the distinction that load theory makes between top-down attention selection settings, which are under voluntary control, and the involuntary allocation of limited-capacity perceptual resources proves useful. On views that equate attention just with the top-down attention settings, the findings that irrelevant stimuli (for which the top-down attention settings are ‘to ignore’) can nonetheless reach awareness in conditions of low load may be taken as evidence for awareness without attention. However, with the mechanistic definition of attention in load theory in which selection will depend on whether capacity limits are reached or not, it is clear that in conditions of low load all stimuli are in fact attended, including those specified as irrelevant. Thus in load theory, there cannot be awareness without the allocation of limited-capacity attention; however, attention cannot be equated with intention or the top-down attentional selection settings.

Of course, following the basic rules of propositional logic, ‘no awareness without attention’ does not imply ‘no attention without awareness’ nor that attention will always lead to awareness. The allocation of attentional resources to stimuli may not always be sufficient to bring them to awareness, and depriving a stimulus of attention may alter its sensory processing at even an unconscious level (we describe direct evidence for this later on in §6). Thus, attention and awareness remain separate in load theory, despite being closely interlinked in many cases.

In this article, we review the contributions of load theory to understand the relationship between attention, awareness and the related neural activity, while including novel data that demonstrate a novel interaction between perceptual load and the fundamental mechanisms of contrast sensitivity.

## 2. Perceptual load and distractor interference measures

**Figure 1.**
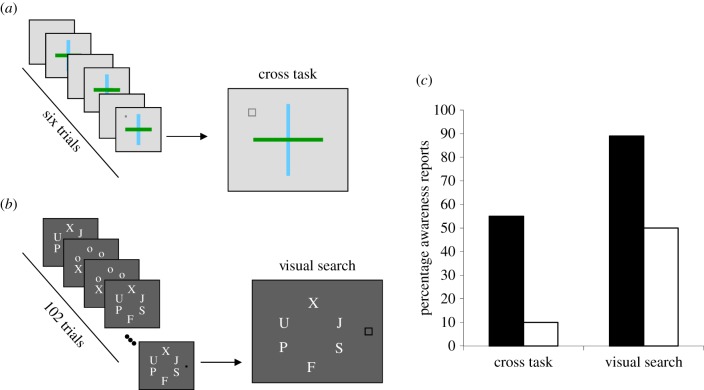
Schematic of the experiment procedures (*a*,*b*) and results (*c*) from [13]. This figure illustrates a load manipulation in which the task varies but the stimuli are identical (*a*) or the set size of heterogeneous items is increased in a random subset of the trials (*b*) across low and high loads. A critical stimulus is added on the last trial. Both load (black bars, low load; white bars, high load) manipulations led to a substantial reduction in awareness reports for this stimulus (*c*). (Online version in colour.)

Early work established the effects of perceptual load on perception somewhat indirectly, using measures of distractor interference on the task reaction time (RT). The level of perceptual load in the task can be increased either by presenting a larger number of heterogeneous items to be processed or by increasing the number and complexity of perceptual operations that the task involves (while keeping the number of stimuli constant across the levels of load, see figure 1*a*,*b*). Both types of perceptual load manipulations were found to reduce distractor interference effects. For instance, in response-competition experiments the extent to which target RT is slowed by the appearance of distractors that are associated with another target response (compared to response-congruent or response-neutral distractors) reflects the cost to distractor processing. Perceptual load was found to significantly reduce distractor response-competition effects [9,14], even when the distractor was presented at fixation [15] as well as negative priming effects (measured for distractors that appear as the target on a subsequent trial, e.g. [16]). Perceptual load was also found to significantly reduce effects of irrelevant attentional capture: the slowing of target RTs in the presence (versus absence) of salient but entirely irrelevant distractors [17]). We note that other types of task load that are not perceptual but instead load top-down processes of cognitive control (e.g. working memory) that are required for active maintenance of the processing priorities in the task can lead to the opposite effect, increasing distractor interference rather than reducing it (owing to reduced control over the task priorities). This contrast clarifies the specificity of the effects on distractor processing to perceptual load *per se* (see [10,11,18] for review of other types of task load).

These effects establish the critical role for perceptual load in determining the efficiency of task performance in the face of distractions. However, they cannot lead to direct conclusions about the effects of perceptual load on conscious perception. The elimination of distractor effects on RT by higher loads might be attributable to reduced distractor intrusions into conscious perception, as the theory predicts, but RT effects are also open to alternative accounts that postulate no role for perceptual load in conscious perception. For example, it is known that priming can result from unconscious processing of the distractors [19] raising the possibility that both the distractor interference effects in low load and their reduction under high load may reflect various degrees of unconscious processing. Note that while response competition and negative priming can be explained in this way, this interpretation fits less well with the demonstrations of reduced attentional capture by entirely irrelevant distractors (e.g. cartoon characters, such as Superman) under higher load. As the cartoons are not associated with task responses, their interference with the task RT cannot be attributed to unconscious priming of any of the letter responses in the search task. The effects of irrelevant capture were also recently found to correlate with conscious reports of mind-wandering [20] in further support of the interpretation that the capture effects reflect intrusions into perceptual awareness, which, by extension, is subjected to modulation by perceptual load (see also Forster & Lavie's [21] demonstration of the modulation of mind-wandering by perceptual load).

Yet another alternative account of the effects of perceptual load on distractor interference suggests that distractors might always enter awareness, regardless of the level of perceptual load. On this account, the reduced distractor interference effects under higher load might reflect their de-prioritization when the task becomes more difficult (although see Lavie & de Fockert's [22], demonstration that increased task difficulty with only minimal increase in perceptual load does not lead to reduced distractor interference effects). Thus, the effects of perceptual load on the RT measures of distractor processing can be attributed to processes that are either earlier (e.g. unconscious priming) or later (e.g. de-prioritization of response) than awareness.

## 3. Perceptual load and neural processing related to awareness

Increased perceptual load is associated with a larger signal in fronto-parietal regions [23–27]. This may reflect a more effective application of the top-down bias in conditions of high load. Importantly, load theory also leads to clear and strong predictions about sensory brain responses to stimuli. Task-irrelevant stimuli should elicit a brain response, even with a top-down bias to ignore them, in tasks involving low perceptual load. By contrast, high perceptual load should significantly reduce the brain response to task-irrelevant stimuli. Numerous neuroimaging studies provided support for these predictions, showing that the level of perceptual load in the task determines the neural response evoked by a wide range of task-irrelevant stimuli. For instance, sensory signals and activity in ventral visual cortex in response to task-irrelevant letters and images of places and objects can be reduced when subjects are engaged in a high perceptual load task [28–31]. Moreover, visual cortex responses evoked by visually salient stimuli (e.g. flickering checkerboards, moving dot displays) are also modulated by the level of perceptual load in a task [32–34]. These modulations can even be found as early as primary visual cortex (V1) and the lateral geniculate nucleus [35,36]. Furthermore, effects of perceptual load were found on temporally early signals [37] and within the first 100 ms of processing [38]. In fact, this last result was obtained with the C1 ERP component which is thought to reflect the initial afference to V1 [37,38], suggesting that perceptual load is influencing the excitability of V1 such that unattended information is being modulated on first arriving in V1.

Clearly then, distractor processing throughout the visual stream depends on the level of perceptual load. For most theories of awareness, such variation in neural processing is a prerequisite for variation in awareness [39–41]; awareness is correlated with the extent to which the stimulus activates both early sensory and category-selective areas of visual cortex [42–49].

However, without direct assessment of their effects on awareness reports, the load modulations of neural responses to distractors described above remain suggestive with respect to the role of perceptual load in awareness. So far, only one of the load neuroimaging studies has included a measure of awareness. Rees *et al.* [32] accompanied their fMRI experiment with a measure of the subjective reports of motion after-effects and found that higher perceptual load not only modulated motion-related activity in V5, but also led to reduced subjective duration of the reported motion after-effect. Evidence that directly ties the effects of perceptual load on the level of visual cortical signal to subjective awareness reports comes from a more recent TMS study. Muggleton *et al.* [50] measured the intensity of magnetic stimulation over area V5 that was required to elicit the subjective percept of a flash of light (in other words the phosphene threshold), while subjects performed a letter search task under different levels of perceptual load (similar to the search task shown figure 1*b*). High perceptual load in the search task resulted in increased phosphene threshold and this was true even when participants made the phosphene report first, before the search response, thus ruling out alternative accounts in terms of a greater likelihood of a memory decay under higher load. In addition, these effects were only found within a time period related to the perceptual processing in the task, thus suggesting the effects were not due to general effects of task difficulty, for example, leading to a more conservative response criterion in the high load. Moreover, the effects of perceptual load on the motion after-effect have been recently replicated in a study that used the criterion-free nulling technique to assess the perception of the motion after-effect [51]. Although the results of these studies are encouraging for our claim that perceptual load is an important determinant of the relationship between attention and awareness, they are confined to the case of visual motion. Some of the other imaging findings described earlier may not necessarily implicate a change in awareness. For example, the demonstrations of modulations of V1 response to irrelevant checkerboards under load may be taken on some accounts [39] to reflect variations of unconscious processing, although note that demonstrations of the critical role of feedback loops between V1 and extrastriate visual areas for perceptual awareness [52] can also explain how these early modulations by load may be tied to changes in conscious perception. The studies reviewed in §4 directly address the effects of perceptual load on subjective awareness reports for a great variety of stimuli and awareness measures.

## 4. Perceptual load and direct measures of subjective awareness

Direct measures of subjective awareness have been used in a range of paradigms: inattentional blindness, change blindness, attentional blink and signal detection. Perceptual load was found to modulate awareness reports in all these paradigms as we review next.

### (a) Load-induced inattentional blindness

Cartwright-Finch & Lavie [13] set out to test directly the effects of perceptual load on subjective awareness reports within the inattentional blindness paradigm [53]. They manipulated perceptual load in a cross task (figure 1*a*) or search task (figure 1*b*) and found substantial effects of perceptual load on the rates of inattentional blindness reports (figure 1*c*). Interestingly, the baseline level of awareness in conditions of low load varied between experiments but the robust modulation by perceptual load remained of similar magnitude.

More recently, a similar result was found for dynamic inattentional blindness displays in which participants tracked moving letters [54]. When the motion-tracking load was higher (letters moving at higher speeds) fewer participants (32% as opposed to 71%) detected an unexpected cross that moved straight across the screen.

Although these findings are a step in the right direction of determining whether perceptual load determines awareness, the retrospective measure of awareness with a surprise question about an unexpected stimulus, raises the possibility that failures to report the presence of the stimulus may reflect, at least in part, rapid forgetting (i.e. ‘inattentional amnesia’; [55]) of a weakly encoded unexpected stimulus. Another possibility is that the inattentional blindness findings reflect a change in the response criterion, such that people may be more reluctant to admit noticing an unexpected stimulus for which there is only a weak trace in conditions of high perceptual load.

Macdonald & Lavie [56] therefore devised a visual search plus detection task in which detection is assessed for an expected stimulus (a meaningless squiggle, appearing in the periphery), thus avoiding the delay involved in processing a surprise question. Moreover, in some experiments, participants made their detection response before the search task response, eliminating the concern about rapid forgetting or poorer encoding into memory during the longer response times found under high load. It also rules out the possibility of de-prioritization of the detection response under high load (cf. the alternative accounts in terms of changes in response selection discussed for the measures of distractor effects on RT in §2). This design also afforded a signal detection analysis so that the effects of perceptual load on perceptual sensitivity *per se* could be assessed independently from any potential effects on the response criterion. The results clearly demonstrated a ‘load-induced blindness’ phenomenon: despite anticipating and actively attempting to detect the peripheral event, participants had lower perceptual sensitivity (*d’*) to the peripheral events when the load of the concurrent task was high than when it was low. Carmel *et al.* [57] extended these findings to show that high perceptual load in visual search reduces sensitivity for the elementary process of detection of a light flicker, presented at fixation.

The findings generalized to a manipulation that changes the processing requirements from feature detection (low load) to discrimination between conjunctions of features (high load) for the very same rapid stream of visual stimuli presented at fixation. Detection sensitivity for a peripheral ‘squiggle’ stimulus was reduced under the high-load condition. Moreover, these effects extended also up to 750 ms following the primary RSVP task stimuli, ruling out the possibility that the reduction was owing purely to sensory competition between the two displays [58]. Importantly, in all these studies, perceptual load effects on detection sensitivity were not accompanied by a change in the response criterion, thus supporting the load theory hypothesis that sensory perceptual processing is reduced in conditions of higher load.

In further support of this conclusion are the findings that the disappearance of a target owing to an artificial scotoma (i.e. the process of filling in with a dynamic noise background) is less likely and takes longer in conditions of high versus low perceptual load [59].

Note that the various forms of load-induced blindness were found regardless of whether the stimulus for which awareness was measured was irrelevant and unexpected [13] or was defined as task-relevant and the participants wilfully attempted to detect it [56,57,59].The effects of perceptual load thus are independent of task-relevance, intention (whether to detect or to ignore), or expectancy. Indeed studies that directly compared relevant and irrelevant distractors of different levels of expectancy [17] report equal modulations by load for both.

### (b) Perceptual load effects on the ‘attentional blink’

Perceptual load has also been shown to increase the magnitude of failures of awareness owing to a form of a psychological refractory period termed the attentional blink (AB): the reduced rates of awareness for the second of two targets presented within half a second in a rapid visual stream. Higher perceptual load in the processing of the first target (e.g. requiring discrimination as opposed to detection in low load or discrimination in the presence of incongruent flanking items versus congruent ones in lower load) leads to a greater rate of AB [60–63]. Moreover, higher perceptual load is thought to cause AB at an earlier perceptual locus compared with the AB in conditions of low perceptual load. For example, although both high and low load targets produced an AB, only higher load eliminated the N400 signature of semantic processing of the second target [61]. Thus, while the locus of the AB effect on awareness is typically thought to be post-perceptual, attributed, for example, to interference with encoding into working memory [64] or with entering into the fronto-parietal ‘global work space’ network [65], higher perceptual load can lead to an earlier locus of AB. These results are consistent with our suggestion that perceptual load effects on inattentional blindness are due to reduced detection sensitivity, and extend the effects of perceptual load to later perceptual processes of sematic identification within a rapid serial stream.

### (c) Perceptual load and awareness of natural scenes and objects of socio-biological significance

Some studies have reported that the gist of a natural scene (e.g. beach versus mountain) appears immune to inattentional blindness [53] and that detection of the presence of an animal in a scene did not suffer from a dual-task cost even under conditions that were shown to impair detection of letters [66]. This type of findings led some researchers to claim that awareness does not require attention (see [67] for discussion). How do we reconcile this with the load theory claim that all perceptual processing is subject to capacity limits and thus depend on selective attention? Furthermore, how can the meaning or gist reach awareness if inattention under high perceptual load reduces early visual processing related to detection sensitivity for elementary visual properties [56], which is presumably necessary for detection of the scene meaning? This apparent conflict can be explained by suggesting that both the gist of a natural scene and objects of high socio-biological significance (e.g. faces) are inherently primed and so require a lower activity threshold to be perceived. Feedback loops between inferior temporal cortex and earlier regions can sensitize the early regions (e.g. striate cortex) to detect patterns consistent with these primed stimuli, allowing them to reach awareness under conditions of reduced availability of attention that typically would preclude awareness for other objects.

However, if all perceptual processing is subjected to capacity limits, as load theory proposes, including stimuli of socio-biological significance, then processing of even these stimuli should be impacted by sufficiently high load. In other words, if high perceptual load reduces both the signal in one area and weakens the strength of vertical connections that mediate feedback loops between areas, then awareness for primed stimuli would also be reduced when the load is high enough. Indirect support for this suggestion comes from the findings that awareness of natural scenes does depend on attention in tasks that appear to involve a higher level of perceptual load (e.g. higher set sizes or more rapid presentation rate compared with the earlier studies; see [68,69]). More direct support for this claim comes from a recent study [70] that varied the level of perceptual load in a motion-tracking task and found substantial reduction in the perception of the natural scene background in conditions of higher perceptual load (involving higher speed compared with the low-load conditions).^2^

Our account helps also to reconcile the findings that change blindness (the failure to detect a change between two images across some form of visual disruption (e.g. a screen flicker or ‘mud splashes’ [71]) is often found for natural scenes as well as for human faces [46,72]. The visual disruption obscures the luminance transient that would normally call attention to the change. Moreover, in the ‘mud splashes' paradigm the disruptions in the form of additional transients, do not overlap with the location of the change. This is suggestive of a role for attention in this phenomenon, (with the disruptions serving to draw attention away from the changes), but it does not speak to the role of perceptual load. However, the natural scenes used in most change blindness experiments are typically rich in detail and so appear to involve a high level of perceptual load. More direct support for the role of perceptual load was found in studies that showed that a higher display set size leads to greater rates of change blindness [73,74]; however, as these studies varied the set size of the stimuli that are the candidates for change their effects might be attributed to increased decision uncertainty in the higher set sizes rather than any change in perceptual awareness. More direct support for the role of perceptual load in awareness or blindness for change comes from a study that assessed change detection for images of faces or places that flanked a letter search display (see [75] for a preliminary report). Perceptual load was varied in the letter search task in a similar manner to that depicted in figure 1*b*. On change trials, one of the face or place images changed to another image from the same category. The pair of successive displays cycled twice (a ‘two-shot’ instead of ‘one-shot’ paradigm) in order to avoid floor level performance. As predicted, greater rates of change blindness occurred during high perceptual load. Note that because perceptual load was varied for a separate letter task, these effects can be more clearly attributed to limited-capacity attention allocation, rather than to increased clutter which may cause low-level visual interference (e.g. lateral masking) or increased decision uncertainty (with greater number of stimuli that could potentially change) in the high-load condition.

## 5. Perceptual load and the contrast response function

The fact that phenomena of subjective blindness under high perceptual load are also associated with neural modulations of primary visual cortex raises the interesting possibility that perceptual load effects might be akin to turning down the contrast of a stimulus. This, then, would be a fundamental limit on awareness; perceptual load may turn down the contrast of an unattended stimulus rendering it less visible. If this were the case, then it could explain the various phenomena of load-induced blindness described above. To investigate this possibility, we examined the effects of perceptual load on the contrast response function (CRF). By plotting the psychometric function relating stimulus contrast to discrimination sensitivity under different levels of load, we can establish how perceptual load might affect visual processing of stimulus contrast. The hypothesis that perceptual load might reduce visual cortex response to contrast would predict a rightward shift in the CRF, so that in conditions of higher perceptual load the stimulus requires a higher level of contrast to be detected compared with conditions of low perceptual load: an effect of reduced contrast gain. Alternatively, the effects of perceptual load might be independent of those of stimulus contrast, in which case high perceptual load might only reduce the level of response gain at each level of contrast (resulting in a lower asymptote and shallower slope of the CRF but no rightward shift).

**Figure 2.**
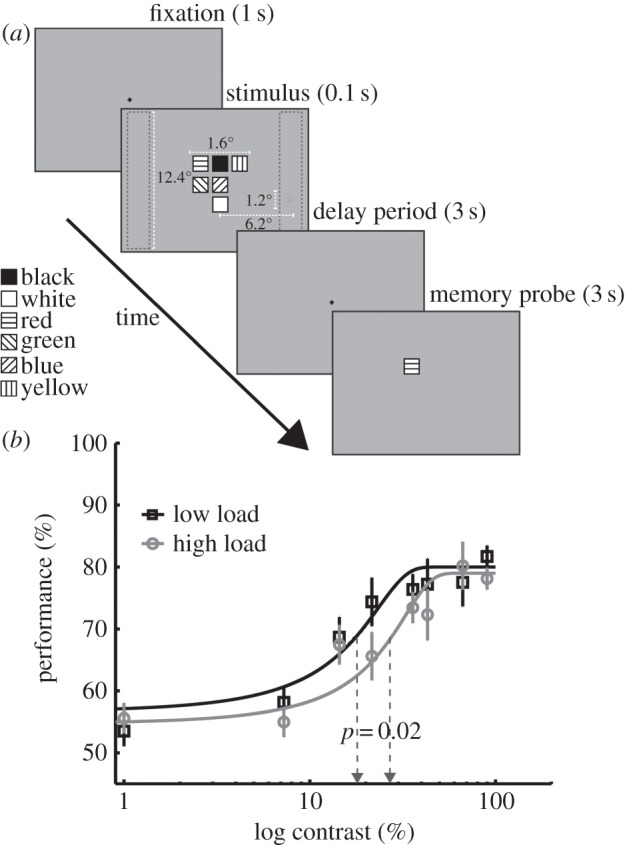
Perceptual load and the CRF. (*a*) Example of high-load displays. (*b*) Psychometric functions for low- (black curve) and high- (grey curve) perceptual load. The estimated contrast threshold parameter for each psychometric function is also shown in dashed vertical lines (contrast threshold yielding half-maximum performance). Each data point represents the mean across participants. Error bars are ±1 s.e.m.

Previous studies of the effects of attention on the CRF have typically assessed the effects of spatial cuing [76,77] but have not varied perceptual load. Of greater relevance to our question are two studies that compared the CRF in single- versus dual-task conditions, which required the participants to perform the contrast detection task concurrently with another task: either an additional oddball detection task [78] or an RSVP task [79]. While they both showed a reduced response gain in the dual (versus single) task conditions only, Huang & Dobkins [79] also found reduced contrast gain in the dual-task conditions. Although differences in the contrast ranges used in the studies may contribute to the difference in results, the arguably more demanding RSVP task used by Huang & Dobkins [79] raises the possibility that high perceptual load would reduce contrast gain. In both of these studies, however, the dual- but not single-task conditions confounded attention with a delay in the response to the contrast task (in order to first accommodate the central task response). Thus, the effects of dual (versus single) task on the CRF may be owing, in part, to the rapid forgetting and deprioritization of the CRF task, in addition to or instead of an effect of perceptual load. Therefore, to assess the effects of perceptual load on the CRF without confounding the effects of deprioritization or rapid forgetting we designed a novel perceptual load task in which participants performed an orientation-discrimination task (for Gabor patches of differing contrasts) while encoding into short-term memory either just the colour feature of a single square (low load) or the conjunction of colour and location for a set of six squares (high load, see figure 2*a*). In this way, the task remained a dual task under both of the load conditions, and perceptual load was varied at the time that the peripheral Gabor patch was presented; however, the participants responded immediately to the orientation task, whereas the effectiveness of the perceptual load manipulation was measured upon the appearance of a memory probe following a delay. Note that as the primary task required continuous maintenance of the memory set throughout the whole trial period, subjects could not just serially perform first the encoding task, then the CRF task. Instead, they clearly had to share attention between the two tasks.

### (a) Method

#### (i) Participants

Fourteen University College London students (aged 20–35 years) were paid for their participation.^3^ All participants had normal or corrected-to-normal vision and no colour blindness.

#### (ii) Stimuli and procedure

Figure 2*a* depicts the stimuli and procedure for this experiment. Trials started with a 1 s presentation of a fixation cross followed by a 100 ms presentation of a stimulus display, which contained a set of one (low load) or six (high load) coloured squares (0.38° × 0.38°) randomly placed on a 3 × 3 grid (1.38° × 1.38°) centred at fixation. Each square was of a different colour, chosen randomly from black (less than 0.01 cd m^−2^), blue (*x* = 0.15, *y* = 0.07; 29.05 cd m^−2^), cyan (*x* = 0.20, *y* = 0.27; 69 cd m^−2^), green (*x* = 0.27, *y* = 0.59; 65.84 cd m^−2^), magenta (*x* = 0.28, *y* = 0.14; 48.20 cd m^−2^), pink (*x* = 0.32, *y* = 0.30; 69.14 cd m^−2^), red (*x* = 0.62, *y* = 0.33; 39.56 cd m^−2^), white (77 cd m^−2^) and yellow (*x* = 0.40, *y* = 0.49; 73.61 cd m^−2^).

The stimulus display also contained a tilted Gabor in the periphery for the orientation-discrimination task. The Gabor patch (sinusoidal grating of 3 cpd enveloped in a Gaussian window, tilted left or right) was presented with equal likelihood within a left or right columnar bar (vertical length: 12.4°; horizontal eccentricity from midline: 6.2°) with the exact location within the columnar bar randomly assigned. The tilt angle of the Gabor patch was individually assessed for each participant with a staircase procedure prior to the main experiment using an accelerated stochastic approximation method obtaining target-contrast estimates that resulted in approximately 75% accuracy rate [81]. In order to capture the full psychometric function, the method of constant stimuli was used; the Gabor contrast was randomly chosen in each trial from a set of eight contrasts (0.1, 7.3, 14.4, 21.6, 35.9, 43.1, 66.5 and 90%) with an equal number of contrasts across all trials.

Following the stimulus display, participants were given up to 2.5 s to respond with their left hand as to whether the Gabor was tilted clockwise (index finger) or counter-clockwise (middle finger), followed by a 500 msec blank screen. Next, a memory probe appeared for up to 3 s (or until response) comprising one coloured square either in the same location as the single item from the memory set (low load) or in any of the memory set locations (high load). Participants indicated with a right-hand response whether the colour of the memory probe matched that of the memory set item that appeared at the same location as the memory probe (index finger indicated ‘same’, middle finger indicated ‘different’, each condition ‘equally likely’). Following an incorrect memory response, ‘WRONG memory response’ appeared above fixation. Responses to the two tasks were not speeded. Participants completed six 64-trial runs (following one practice run), resulting in a total of 384 trials (192 trials per load condition). The perceptual load condition was blocked in eight-trial blocks (counterbalanced in an ABBABAAB fashion) within each run.

### (b) Results and discussion

Accuracy rates in the visual short-term memory task decreased significantly from the low- (*M* = 95%, s.d. = 3%) to the high- (*M* = 64%, s.d. = 4%) load condition, *t*_13_ = 29.65, *p* < 0.001, *d* = 1.91, indicating that the high-load condition was more difficult. Moreover, a two-way ANOVA on visual short-term memory accuracy rates as a function of load (low, high) and orientation-discrimination accuracy (correct, incorrect) showed no effect of orientation-discrimination accuracy nor an interaction (*F* < 1 for both), thus confirming that subjects prioritized the visual short-term memory encoding over the orientation-discrimination as instructed.

To assess whether the effects of perceptual load on visual detection are consistent with contrast gain or response gain, the data from each participant were fitted to the Weibull contrast response model. *ψ*(*x*; *α*, *β*, *γ*, *λ*) = *γ* + (*1 – *γ* – *λ*) *F*(*x*; *α*, *β*), where *x* is the stimulus contrast, *α*, *β*, *γ*, *λ* are the fitted model parameters which determine the shape of the psychometric function, and *F* is the Weibull function: *F*(*x*; *α*, *β*) = 1 – exp(–(*x*/*α*)*^*β*^*), with *x* ∈ (−∞,+∞), *α* ∈ (−∞, + ∞), *β* ∈ (−∞, +∞). The contrast threshold (*α*, alpha), the slope (*β*, beta) and the asymptote (*λ*, lambda) of the CRF were left to vary freely and estimated separately for the low- and high-load conditions. Gamma (*γ*)* represented the chance level and was set at 0.50.

Fits were performed using maximum-likelihood estimation. Goodness-of-fit was assessed with deviance scores, which were calculated as the log-likelihood ratio between a fully saturated model and the data model. This analysis confirmed good fits in all participants, as indicated by cumulative probability estimates of the obtained deviance scores (all *p*-values < 0.05).

Figure 2*b* shows the group-average psychometric functions and their Weibull fits for the low- and high-load conditions. A comparison of the estimated individual contrast thresholds (α) between low and high load confirmed a significant increase in the contrast threshold from the low- (*M* = 18%, s.d. = 8%) to high-load condition (*M* = 27%, s.d. = 13%), *t*_13_ = 2.64, *p* = 0.023, *d* = 0.79 (figure 2*b*). Importantly, the same analysis did not reveal any significant differences on the estimated asymptotes (low load, *M* = 80%, s.d. = 7%; high load, *M* = 79%, s.d. = 9%, *t*_13_ = 0.20, *p* = 0.85, *d* = 0.07) or the slope (low load, Mdn = 4.21, s.d. = 42.54; high load, Mdn = 4.41^3^, s.d. = 46.08), *t*_13_ = −0.27, *p* = 0.79, *d* = 0.08; of^4^ the psychometric function between the two conditions.

These results demonstrate that high perceptual load shifts the CRF to the right without affecting the slope and the asymptote of the psychometric function. This finding is consistent with the predictions of the contrast gain model and indicates that perceptual load interacts interchangeably with contrast. Note that as in many of the previous load studies reviewed above, our manipulation of load in a colour- and location-based task did not change the feature relevance of the CRF task (which concerned contrast and orientation). This suggests that our effects cannot be accounted for in terms of reduced feature-based attention (which should only lead to an effect on response gain, see [78]), but instead are caused by the increased demand on perceptual capacity in the high-load conditions.

Our findings that perceptual load can reduce the contrast gain, an effect equivalent to a reduction in the effective stimulus contrast, suggest a viable mechanism for the various effects of load-induced blindness (§4) in terms of reduced neural sensitivity to contrast, in conditions of high load.

## 6. Perceptual load and unconscious processing

It is useful at this point to make a clear distinction between the possibility that attention serves as a gateway specifically for awareness (as defined in §1*a* as a phenomenological perceptual experience that is accessible to conscious report) and the possibility that attention is the gateway to all sensory processing of stimuli from its very early stages, including those that are unconscious.

Although all the studies reviewed so far show that high perceptual load reduces the level of perceptual awareness, and as such are open to both interpretations, in load theory the effects of perceptual load should not be confined to awareness. The competition for limited-capacity perceptual and neural representation resources should not be restricted to just conscious representations.

To address directly the effects of perceptual load on unconscious processing, Bahrami *et al.* [82] manipulated perceptual load in an RSVP task presented at fixation and measured V1 responses to stimuli that were rendered effectively invisible with the continuous flash suppression (CFS) method [83]. Despite the fact that the stimuli remained invisible under both load conditions, V1 responses to these invisible gratings were significantly reduced with higher perceptual load in the task. Bahrami *et al.* [84] have also demonstrated that high perceptual load reduces orientation adaptation for orientation stimuli that were again suppressed from conscious awareness with the CFS method.

Previous attempts to investigate whether unconscious processing of orientation depends on attention lead to discrepant results; for example, a manipulation of spatial attention was found to either reduce [85] or have no effect on [86] orientation adaptation for stimuli rendered unconscious during adaption. These inconsistencies underscore the importance of considering perceptual load in assessing the effects of attention. The earlier studies simply asked participants either to attend to or away from the unseen adaptor. Perceptual load theory predicts that such requests will not be sufficient to modulate processing of the unseen stimulus. Instead, effects of attention on unseen (or seen) ignored stimuli will only be observed when resources are sufficiently engaged by another task or stimulus and thus unavailable to the stimulus in question. By varying the perceptual load of a task, Bahrami *et al.* [82,84] showed clear effects of attention on unconscious processing. Indeed, Bahrami *et al.* [84] used the same CFS method as Kanai *et al.* [86] to render the orientation stimuli invisible during adaptation; thus the contrasting findings can be safely attributed to the manipulation of perceptual load rather than other factors, for example, the effectiveness of the method used for suppression from consciousness.

These findings challenge some proposals that equate attention with awareness [87,88] as well as those suggesting that attention acts as the gateway to awareness (e.g. [89]) or can only act on conscious representations to allow them to be reported [90]. They clarify that perceptual processing has limited capacity, even at early levels of representations that remain unconscious, and emphasize the importance of considering perceptual load throughout the perceptual processing stream from unconscious to conscious levels.

## 7. Summary and conclusion

Load theory resolved the enduring debate between early and late selection views as to whether attention excludes irrelevant information before or after perceptual awareness. Instead of attention having a fixed locus, the theory argues that awareness depends on the availability of limited-capacity attention. High perceptual load exhausts capacity, whereas low perceptual load leaves ‘spare’ capacity, resulting in full perceptual awareness of both relevant and irrelevant information. Thus, although the allocation of limited-capacity attention is a necessary condition for awareness, attention and perceptual awareness are selective only in conditions of high perceptual load. The evidence we discuss includes the effects of load on the level of distractor intrusions, neuroimaging reports showing extensive modulations of the neural response to ignored stimuli throughout visual cortex (including primary cortex, subcortical pathways, early response components, such as C1 and responses to stimuli that remain unconscious) as well as behavioural reports of ‘load-induced blindness’. These behavioural reports were found in various tasks measuring awareness, and a measure of the effects of load on the CRF suggests that perceptual load effects can be equivalent to a reduction in the effective contrast of a stimulus. Thus, the effects of load-induced blindness could be explained in terms of reduced neural sensitivity to contrast, which appear analogous to load dimming the light. The results converged across the different paradigms, some of which used a single task and assessed the effects of load on irrelevant processing (as was the case for the distractor and imaging paradigms, as well as the traditional inattentional blindness tasks), while others (e.g. those using direct measures of awareness reports) used a dual-task design and assessed the effects of load on task-relevant processing. Thus, the effects of perceptual load on information processing apply across the board and the convergence across very different paradigms rules out any alternative accounts in terms of task-specific factors.

Overall, perceptual load has been shown to influence the degree of processing related to perceptual awareness across multiple stages of the visual system, from the very early sensory processing stages (including those remaining unconscious) to those that have a profound effect on visual awareness.

## Endnotes

1 At this point, it is perhaps important to clarify that load theory does not suggest a serial two-stage model whereby attentional allocation to the relevant information happens first followed by a spillover of remaining capacity (cf. [12]). Instead, the same outcome can be achieved with a simultaneous parallel processing model in which a limited capacity is allocated in parallel to all stimuli with their priority reflected in different processing weights.

2 Note that fine-grained variations in load are necessary to investigate an interesting prediction of our claim: some levels of load would be sufficient to exhaust capacity for the non-primed objects, whereas only higher levels of load would be sufficient to exhaust capacity for the primed objects. This should be an interesting future research avenue.

3 Data available from the Dryad digital repository: http://doi.org/10.5061/dryad.h8t06 [80].

4 Medians of the individual mean slopes are reported owing to one outlier subject with an outlier slope value that would have skewed the overall mean.
